# Development and validation of a nomogram integrating marital status for 5-year overall survival of chondrosarcoma: a population-based study

**DOI:** 10.1007/s12672-024-01020-1

**Published:** 2024-05-16

**Authors:** Chengxin Xie, Ruiyuan Jiang, Chenglong Wang, Xinhuan Lei, Kaicheng Lu, Hua Luo

**Affiliations:** 1 Department of Orthopedics, Taizhou Hospital of Zhejiang Province Affiliated to Wenzhou Medical University, Linhai, 317099 China; 2 Shandong First Medical University, Jinan, 250021 China; 3 Department of Graduate Student, Zhejiang University of Chinese Medicine, Hangzhou, 310000 China; 4 Zhejiang Cancer Hospital, Hangzhou Institute of Medicine (HIM), Chinese Academy of Sciences, Hangzhou, 310022 China; 5 Department of Graduate Student, Faculty of Chinese Medicine Science, Guangxi University of Chinese Medicine, Nanning, 530022 China

## Abstract

**Objectives:**

The objective of this study was to evaluate the influence of marital status on overall survival (OS) and develop a nomogram for predicting 5-year OS in chondrosarcoma (CHS) patients.

**Methods:**

We utilized the Surveillance, Epidemiology, and End Results (SEER) database to identify CHS patients diagnosed between 2010 and 2018. Survival rates were calculated using Kaplan–Meier analysis. Prognostic factors were identified through univariate and multivariate analyses. An independent cohort was used for external validation of the nomogram. Performance evaluation of the nomogram was conducted using Harrell's concordance index (C-index), calibration plot, and decision curve analysis (DCA).

**Results:**

In the SEER cohort, Kaplan–Meier analysis showed significant differences in OS among CHS patients with different marital statuses (P < 0.001), with widowed patients having the lowest OS. In terms of gender, there were significant survival differences based on marital status in females (P < 0.001), but not in males (P = 0.067). The OS of married and single females is significantly higher than that of married (P < 0.001) and single male (P = 0.006), respectively. Kaplan–Meier curves showed no significant difference in OS between groups stratified by either gender or marital status in the external cohort. Univariate and multivariate analyses confirmed that age at diagnosis, gender, marital status, tumor size, histological type, tumor grade, SEER stage, and surgery were independent prognostic factors for OS. The nomogram demonstrated high internal and external validation C-indexes of 0.818 and 0.88, respectively. Calibration plots, DCA curve, and Kaplan–Meier curve (P < 0.001) confirmed the excellent performance and clinical utility of the nomogram.

**Conclusions:**

Marital status was an independent factor influencing OS in CHS patients, with widowed patients having the worst prognosis. The OS of both married and single females is significantly higher than that of their male counterparts. However, these findings require further validation in a large independent cohort. While the contribution of marital status on predicting OS appears modest, our nomogram accurately predicted 5-year OS and identified high-risk groups, providing a valuable tool for clinical decision-making.

**Supplementary Information:**

The online version contains supplementary material available at 10.1007/s12672-024-01020-1.

## Introduction

Chondrosarcoma (CHS) is a heterogeneous malignant cartilaginous tumor with diverse morphological features and clinical behaviors, accounting for approximately 20% of all primary malignant bone tumors [1]. CHS is the second most common bone malignancy after osteosarcoma, with the primary conventional subtype representing over 90% of cases [2]. Treatment of CHS primarily relies on surgical resection as radiotherapy and chemotherapy have limited efficacy on conventional CHS [2, 3].

Previous studies on prognostic factors of CHS have primarily focused on age, histologic subtype, pathological grade, tumor size, distant metastases, and therapeutic regimen [4–6]. Recently, there has been increased attention to the impact of marital status on CHS prognosis since it was identified as an independent prognostic factor for cancer-specific survival [7–9]. Gao et al. [7] reported that widowed patients had a higher risk of CHS cancer-specific mortality. Li et al. [8] and Hoang et al. [9] found that married patients were associated with a higher risk of distant metastasis. However, previous studies have shown that married cancer patients often have better survival outcomes [10–15]. Consequently, further investigation is necessary to clarify the relationship between marital status and CHS prognosis.

Nomograms are reliable statistical prediction tools that offer visualization and accuracy, incorporating various factors such as pathological variables, therapeutic regimens, and demographic variables to predict prognosis. Several nomograms have been developed to predict survival in CHS patients [16–21]. However, these nomograms commonly overlook marital status as a predictor for CHS survival. In this study, we aimed to investigate the effects of marital status, identify independent prognostic factors, and develop a nomogram for 5-year OS in CHS patients.

## Methods

### Data source and patient selection

We obtained data from the Surveillance, Epidemiology, and End Results (SEER) database, which is sponsored by the US National Cancer Institute and records cancer incidence and survival data (https://seer.cancer.gov/). We selected CHS patients diagnosed between January 1, 2010, and December 31, 2018, using SEER Stat software version 8.3.6. Patients were identified based on the International Classification of Diseases for Oncology Third Edition (ICD-O-3) using the morphological codes: 9220 (CHS not otherwise specified (NOS)), 9221 (juxtacortical CHS), 9230 (malignant chondroblastoma), 9231 (myxoid CHS), 9240 (mesenchymal CHS), 9242 (clear cell CHS), and 9243 (dedifferentiated CHS). The site recodes were limited to bones and joints. We applied exclusion criteria, including patients below 18 years old, unknown marital status and domestic partner, unknown race, lack of positive histology, not the first malignant primary indicator, unknown survival time, unknown cancer-directed surgery, and incomplete follow-up. The SEER database is a publicly accessible resource, therefore, obtaining ethics approval and informed consent was not applicable to this cohort.

For external validation, we established a validation cohort using electronic medical records of CHS patients from Zhejiang Cancer Hospital (ZJCH) diagnosed between June 1, 2014, and May 31, 2017. Inclusion criteria included a minimum follow-up duration of 5 years, treatment received solely at ZJCH, age at diagnosis of 18 years or older, bone and joint CHS, and complete medical records. Any information that could lead to participant identification was removed. The study was approved by the licensing committee of Zhejiang Cancer Hospital (Project ID: IRB-2021-31), and informed consent was not obtained as the data was analyzed anonymously. All methods adhered to relevant guidelines and regulations.

### Data collection

The variables collected for the SEER cohorts were as follows: marital status, age at diagnosis, gender, race, tumor location, laterality, tumor size, histology type, tumor grade, SEER stage, total number of in situ/malignant tumors (TNT), surgery of the primary site, radiotherapy, chemotherapy, survival months, and vital status. Marital status was classified as married, separated, divorced, single, and widowed. For the purposes of this study, we grouped separation and divorced into one category. The patient’s race was classified as white, black, or other. The tumor location was categorized into three groups: extremities (bones of the upper and lower extremities), axial (vertebral columns, ribs, sternum, clavicle, and pelvic bones), and other (mandible and bones of the skull or face). Laterality was divided into four groups: non-paired, left, right, and bilateral. Tumor size was categorized into three levels using previously reported cutoff points: < 60 mm, 60–140 mm, and > 140 mm [16]. The stage was coded as localized, regional, and distant according to the SEER program. The endpoint of the study was OS, defined as the time from diagnosis to death for any reason.

### Statistical analysis

Baseline demographic and clinicopathological characteristics were compared using Pearson's chi-square tests and Fisher's exact tests. Kaplan–Meier curves were utilized to estimate and compare OS. Univariate and multivariate Cox regression analyses were conducted to identify prognostic factors. The nomogram was constructed using variables that were found to be significant in the multivariate Cox models. The performance of the nomogram was assessed by C-index and calibration plot. The clinical applicability of the nomogram was assessed using decision curve analysis (DCA) and Kaplan–Meier curves. All statistical analyses and chart formation were performed using IBM SPSS 21.0, R project 4.1.3, and X-tile 3.6.1. All tests were two-sided, and P-values less than 0.05 were considered statistically significant.

## Results

### Baseline characteristics

A total of 1,125 eligible CHS patients, with 45.4% being female, were included from the SEER database. The distribution of these patients across different marital statuses is presented in Table 1. Among the patients, 724 (64.4%) were married, 92 (8.2%) were divorced/separated, 253 (22.5%) were single, and 56 (5%) were widowed. The Chi-square test revealed significant differences in several variables among the different marital statuses, including age at diagnosis (P < 0.001), gender (P < 0.001), race (P < 0.001), histological type (P < 0.001), tumor grade (P = 0.003), and surgery (P = 0.002).

**Table 1:** Baseline characteristics of SEER cohort based on different marital status

Variables	Overall n (%)	Married n (%)	Div/Sep^a^ n (%)	Single n (%)	Widowed n (%)	P-value
Overall	1125(100)	724(64.4)	92(8.2)	253(22.5)	56(5.0)	
**Age (years)**						< 0.001
≤ 40	282(25.1)	146(51.8)	14(5.0)	122(43.3)	0(0)	
41–60	464(41.2)	316(68.1)	43(9.3)	96(20.7)	9(1.9)	
> 60	379(33.7)	262(69.1)	35(9.2)	35(9.2)	47(12.4)	
**Gender**						< 0.001
Female	511(45.4)	311(60.9)	53(10.4)	105(20.5)	42(8.2)	
Male	614(54.6)	413(67.3)	39(6.4)	148(24.1)	14(2.3)	
**Race**						< 0.001
White	987(87.7)	653(66.2)	83(8.4)	203(20.6)	48(4.9)	
Black	86(7.6)	35(40.7)	7(8.1)	37(43.0)	7(8.1)	
Other	52(4.6)	36(69.2)	2(3.8)	13(25.0)	1(1.9)	
**Tumor location**						0.422
Extremities	585(52.0)	377(64.4)	53(9.1)	120(20.5)	35(6.0)	
Axial	448(39.8)	290(64.7)	32(7.1)	109(24.3)	17(3.8)	
Other	92(8.2)	57(62.0)	7(7.6)	24(26.1)	4(4.3)	
**Laterality**						0.593
Non-paired	208(18.5)	136(65.4)	16(7.7)	46(22.1)	10(4.8)	
Left	450(40.0)	289(64.2)	41(9.1)	105(23.3)	15(3.3)	
Right	455(40.4)	290(63.7)	35(7.7)	100(22.0)	30(6.6)	
Bilateral	12(1.1)	9(75.0)	0(0)	2(16.7)	1(8.3)	
**Tumor size (mm)**						0.566
< 60	487(43.3)	324(66.5)	38(7.8)	100(20.5)	25(5.1)	
60–140	495(44.0)	316(63.8)	40(8.1)	118(23.8)	21(4.2)	
> 140	143(12.7)	84(58.7)	14(9.8)	35(24.5)	10(7.0)	
**Histological type**						< 0.001
NOS^b^	910(80.9)	583(64.1)	68(7.5)	221(24.3)	38(4.2)	
Myxoid	75(6.7)	49(65.3)	5(6.7)	17(22.7)	4(5.3)	
Dedifferentiated	113(10.0)	74(65.5)	18(15.9)	7(6.2)	14(12.4)	
Other	27(2.4)	18(66.7)	1(3.7)	8(29.6)	0(0)	
**Tumor grade**						0.003
Low (I–II)	856(76.1)	545(63.7)	61(7.1)	211(24.6)	39(4.6)	
High (III– IV)	269(23.9)	179(66.5)	31(11.5)	42(15.6)	17(6.3)	
**SEER stage**						0.056
Localized	631(56.1)	411(65.1)	47(7.4)	152(24.1)	21(3.3)	
Regional	400(35.6)	250(62.5)	35(8.8)	86(21.5)	29(7.3)	
Distant	94(8.4)	63(67.0)	10(10.6)	15(16.0)	6(6.4)	
**TNT**^**c**^						0.064
1	1034(91.9)	660(63.8)	85(8.2)	241(23.3)	48(4.6)	
2 or 3	91(8.1)	64(70.3)	7(7.7)	12(13.2)	8(8.8)	
**Surgery**						0.002
No	75(6.7)	41(54.7)	3(4.0)	21(28.0)	10(13.3)	
Yes	1050(93.3)	683(65.0)	89(8.5)	232(22.1)	46(4.4)	
**Radiotherapy**						0.644
No	998(88.7)	641(64.2)	79(7.9)	229(22.9)	49(4.9)	
Yes	127(11.3)	83(65.4)	13(10.2)	24(18.9)	7(5.5)	
**Chemotherapy**						0.188
No	1036(92.1)	661(63.8)	84(8.1)	241(23.3)	50(4.8)	
Yes	89(7.9)	63(70.8)	8(9.0)	12(13.5)	6(6.7)	

^a^Divorced/separated

^b^Not otherwise specified

^c^Total number of in situ/malignant tumors

For the validation cohort, a total of 116 CHS patients, with 45.7% being female, from ZJCH met the inclusion criteria. The baseline characteristics of patients in both the training and validation cohorts are presented in S1 Table.

### Kaplan–Meier analysis

**Fig. 1:**
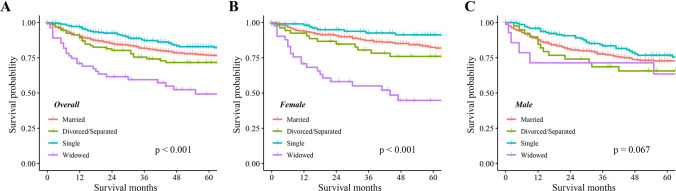
Kaplan–Meier survival curves of overall survival in CHS patients of different marital status. **A** Overall. **B** Female. **C** Male

As shown in Fig. 1A, the result demonstrates a significant difference in OS among the different marital statuses (P < 0.001), with widowed patients experiencing the lowest OS. To further evaluate the impact of marital status on OS according to gender, we analyzed the data as shown in Fig. 1B and C. Among females, there was a significant difference in OS among different marital statuses (P < 0.001), while among males, the difference was not statistically significant (P = 0.067).

**Fig. 2:**
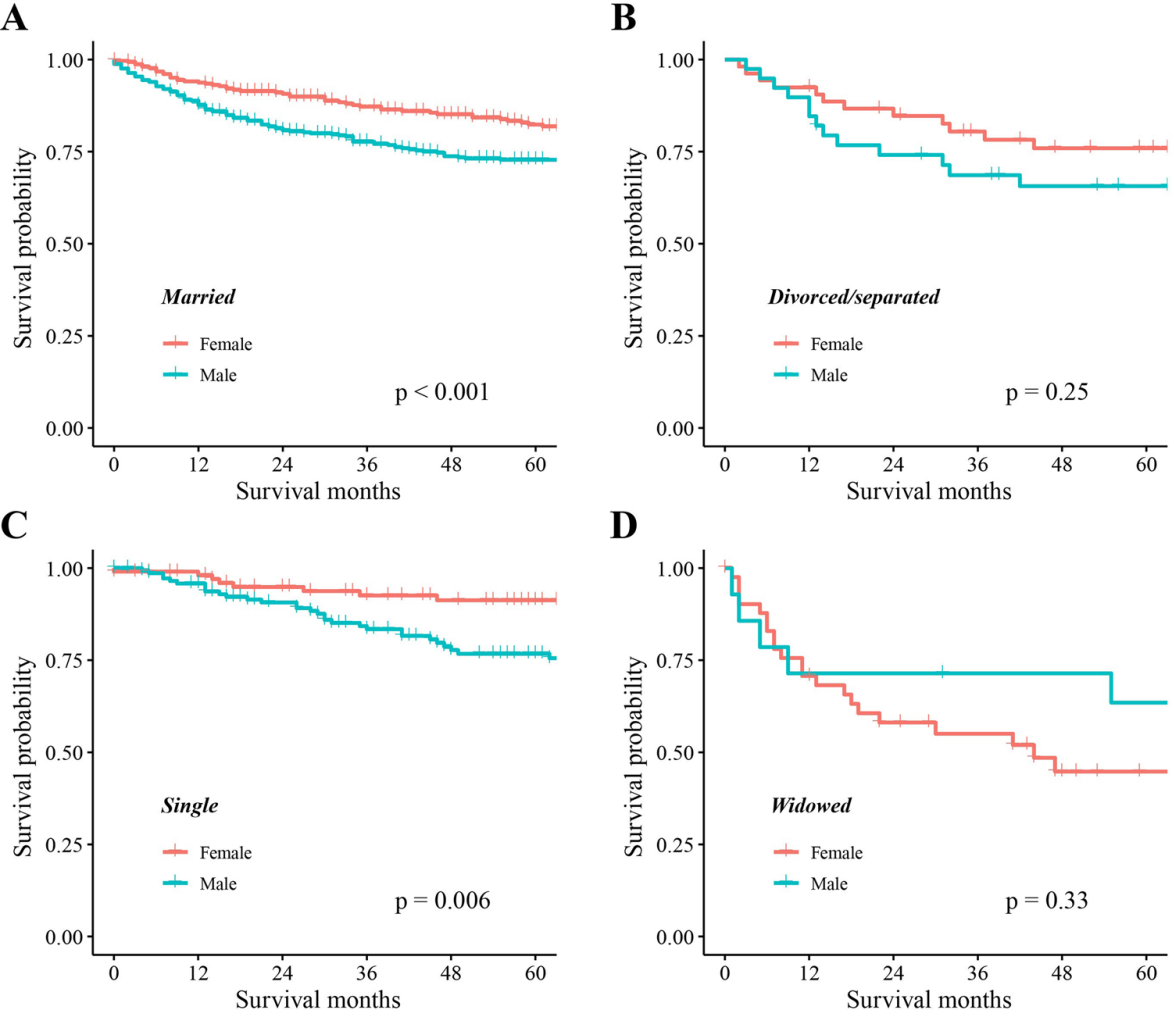
Kaplan–Meier survival curves of overall survival in each marital status with different gender. **A** Married. **B** Divorced/separated. **C** Single. **D** Widowed

Additionally, we examined the OS differences between females and males within each marital status group. As depicted in Fig. 2, the OS of married females was significantly higher than that of males (P < 0.001), and the same was observed for single females (P = 0.006). However, there were no significant differences in OS between females and males among the divorced/separated group (P = 0.25) and the widowed group (P = 0.33).

**Fig. 3:**
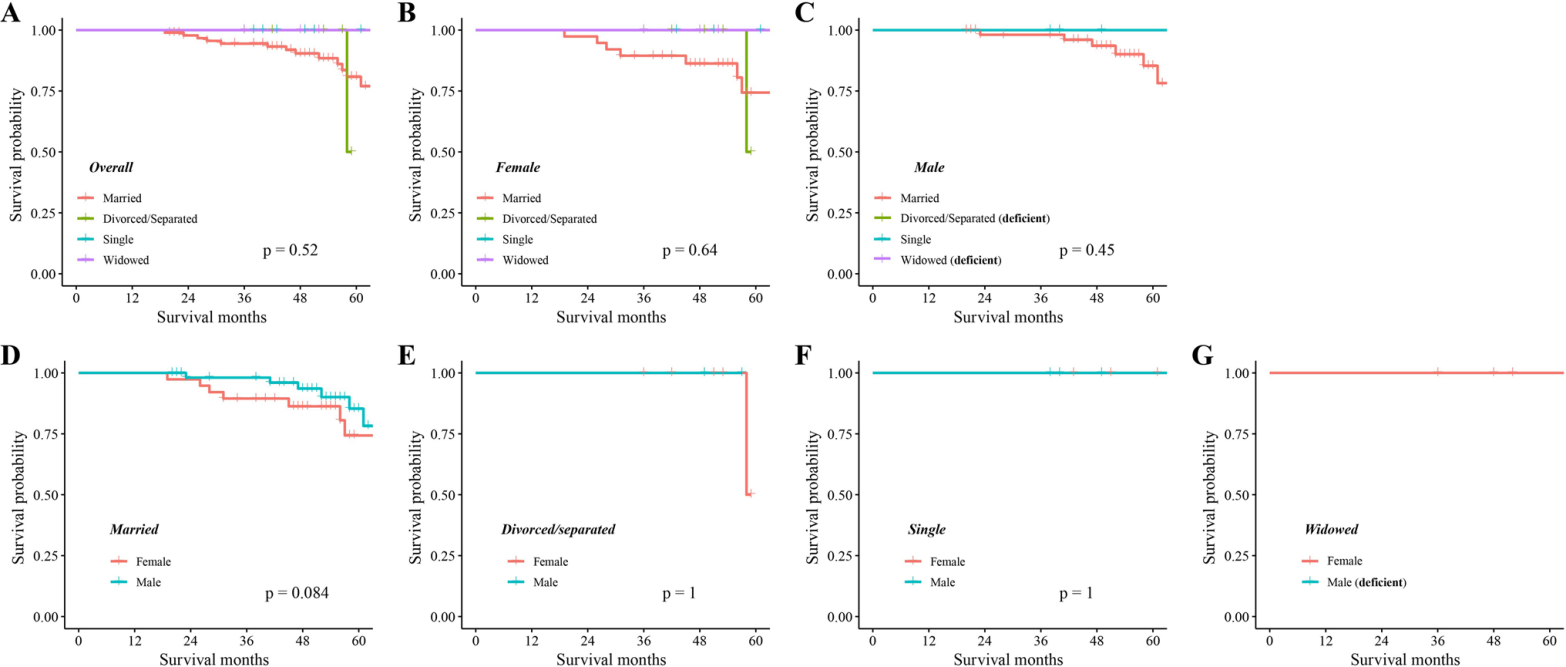
Kaplan–Meier survival curves of overall survival in CHS patients (validation cohort). Grouped by marital status: **A** Overall, **B** Female, and **C** Male. Grouped by gender: **D** Married, **E** Divorced/separated, **F** Single, and **G** Widowed. *Unable to generate a survival curve when the sample size is less than 3 (marked as**** deficient)***

To further verify the importance of marital status on OS, we also performed Kaplan–Meier analysis in the validation cohort. As shown in Fig. 3, the results demonstrate that there was no significant difference in OS between groups stratified by either gender or marital status (all P > 0.05).

### Prognostic factors

A univariate Cox regression analysis was performed using the SEER patient dataset (training cohort). The analysis revealed that several variables were statistically significant for OS in CHS patients, including age at diagnosis (P < 0.001), gender (P < 0.001), marital status (P < 0.001), tumor size (P < 0.001), histological type (P < 0.001), tumor grade (P < 0.001), SEER stage (P < 0.001), surgery (P < 0.001), radiotherapy (P < 0.001), and chemotherapy (P < 0.001). These results can be found in Table 2.

**Table 2:** Univariate and multivariate Cox regression analysis of OS in the training cohort

Variables	Univariate analysis	Multivariate analysis	
	P value	HR^d^ (95%CI^e^)	P value
Age (years)	< 0.001		
≤ 40		Reference	
41–60		2.263(1.507–3.399)	< 0.001
> 60		3.429(2.251–5.223)	< 0.001
Gender	< 0.001		
Female		Reference	
Male		1.694 (1.323 – 2.17)	< 0.001
Marital status	< 0.001		
Married		Reference	
Div/Sep^a^		1.179(0.793–1.751)	0.416
Single		0.932(0.665–1.307)	0.684
Widowed		1.925(1.279–2.898)	0.002
Race	0.4	NI^f^	
White			
Black			
Other			
Tumor location	0.7	NI^f^	
Axial			
Extremities			
Other			
Laterality	0.2	NI^f^	
Non-paired			
Left			
Right			
Bilateral			
Tumor size (mm)	< 0.001		
< 60		Reference	
60–140		1.165(0.889–1.526)	0.269
> 140		1.568(1.105–2.226)	0.012
Histological type	< 0.001		
NOS^b^		Reference	
Myxoid		0.777(0.485- 1.247)	0.296
Dedifferentiated		2.694(1.882–3.858)	< 0.001
Other		1.318(0.522–3.331)	0.559
Tumor grade	< 0.001		
Low (I–II)		Reference	
High (III– IV)		1.402(1.033–1.904)	0.03
SEER stage	< 0.001		
Localized		Reference	
Regional		1.962 (1.493–2.578)	< 0.001
Distant		5.581(3.837–8.116)	< 0.001
TNT^c^	0.09	NI^f^	
1			
2 or 3			
Surgery	< 0.001		
No		Reference	
Yes		0.504(0.35–0.725)	< 0.001
Radiotherapy	< 0.001		
No		Reference	
Yes		1.184(0.87–1.612)	0.284
Chemotherapy	< 0.001		
No		Reference	
Yes		1.229(0.867–1.743)	0.247

^a^Divorced/Separated

^b^Not otherwise specified

^c^Total number of in situ/malignant tumors

^d^Hazard ratio

^e^Confidence interval

^f^Not included

Further analysis was done using multivariate Cox regression models to identify independent prognostic factors for OS in CHS patients. Following variables were independent prognostic factors for OS in CHS patients including age at diagnosis, gender, marital status, tumor size, histological type, tumor grade, SEER stage, and surgery (Table 2). Regarding marital status, a statistically significant difference was observed only in widowed patients compared to married patients (HR = 1.925, 95%CI: 1.279 to 2.898, P = 0.002).

### Nomogram construction

**Fig. 4:**
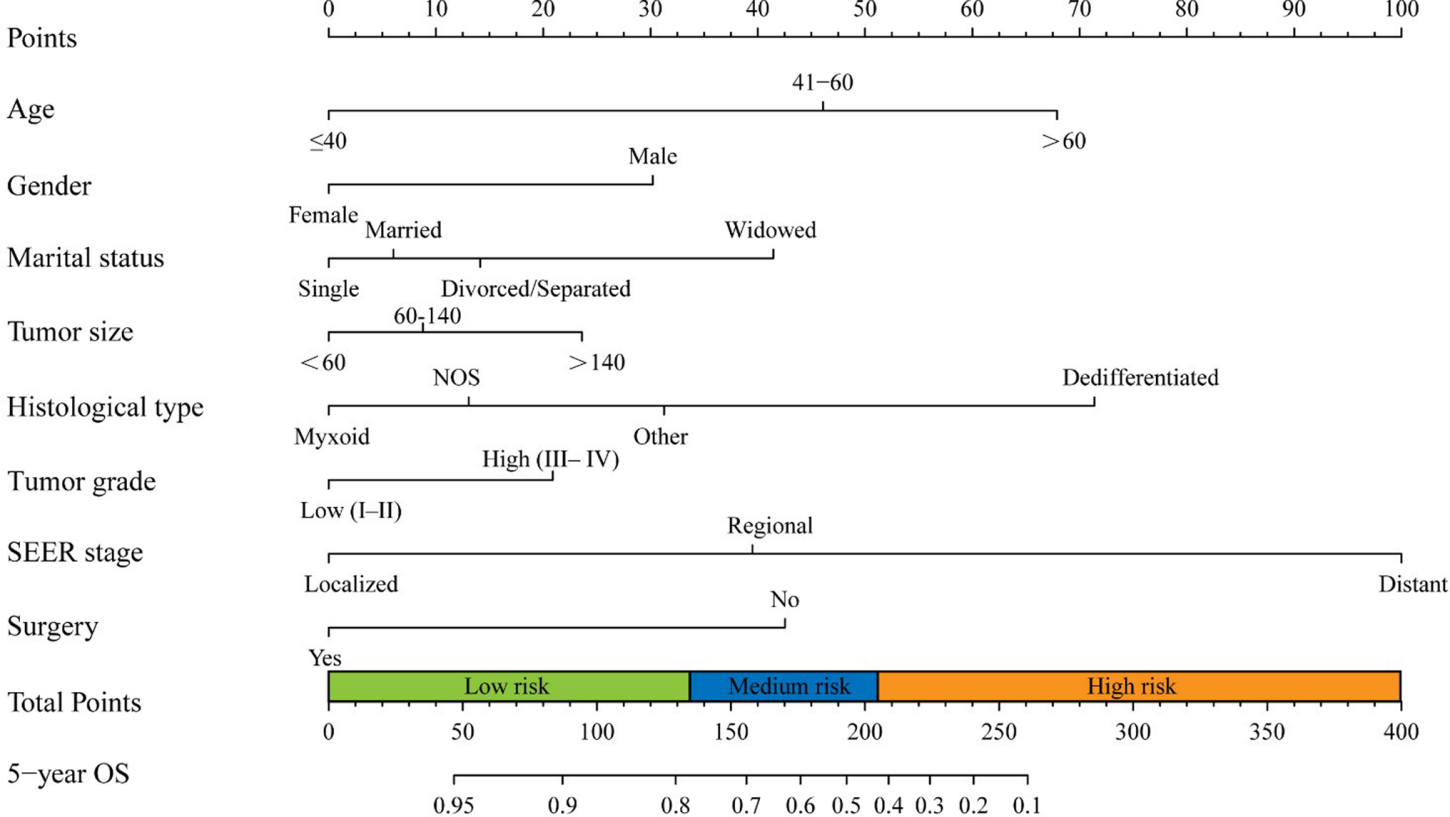
Nomogram for 5-year OS in CHS patients

The coefficients obtained from the multivariate Cox regression model were used to construct a nomogram for predicting 5-year OS. The relationship between the variables' hazard ratios (HR) and their corresponding points allocation on the nomogram can be observed by comparing Table 2 with Fig. 4. Notably, variables with higher HR values are associated with a greater point allocation on the nomogram. The nomogram comprises eight variables, and each subgroup variable is assigned a score ranging from 0 to 100 based on its contribution (see S2 Table). These scores are then added to the scores corresponding to the respective variables to calculate the total scores on the bottom scales of the nomogram. The total scores are further transformed to predict the corresponding 5-year OS probability. Figure 4 illustrates that SEER stage contributes the most to predicting 5-year OS, followed by histological type, age, surgery, marital status, gender, tumor size, and tumor grade. In addition, we constructed a nomogram excluding the marital status factor (see S1 Fig).

### Nomogram validation

The nomogram was subjected to both internal and external validation. The C-index values were calculated to evaluate the performance of the nomogram in predicting 5-year OS. In the training cohort, the nomogram achieved a C-index value of 0.818, while in the external validation cohort, the C-index value was 0.88. These values indicate good discriminatory ability of our nomogram. In addition, the nomogram excluding marital status exhibited C-index values of 0.815 and 0.891 through internal and external validation, respectively.

**Fig. 5:**
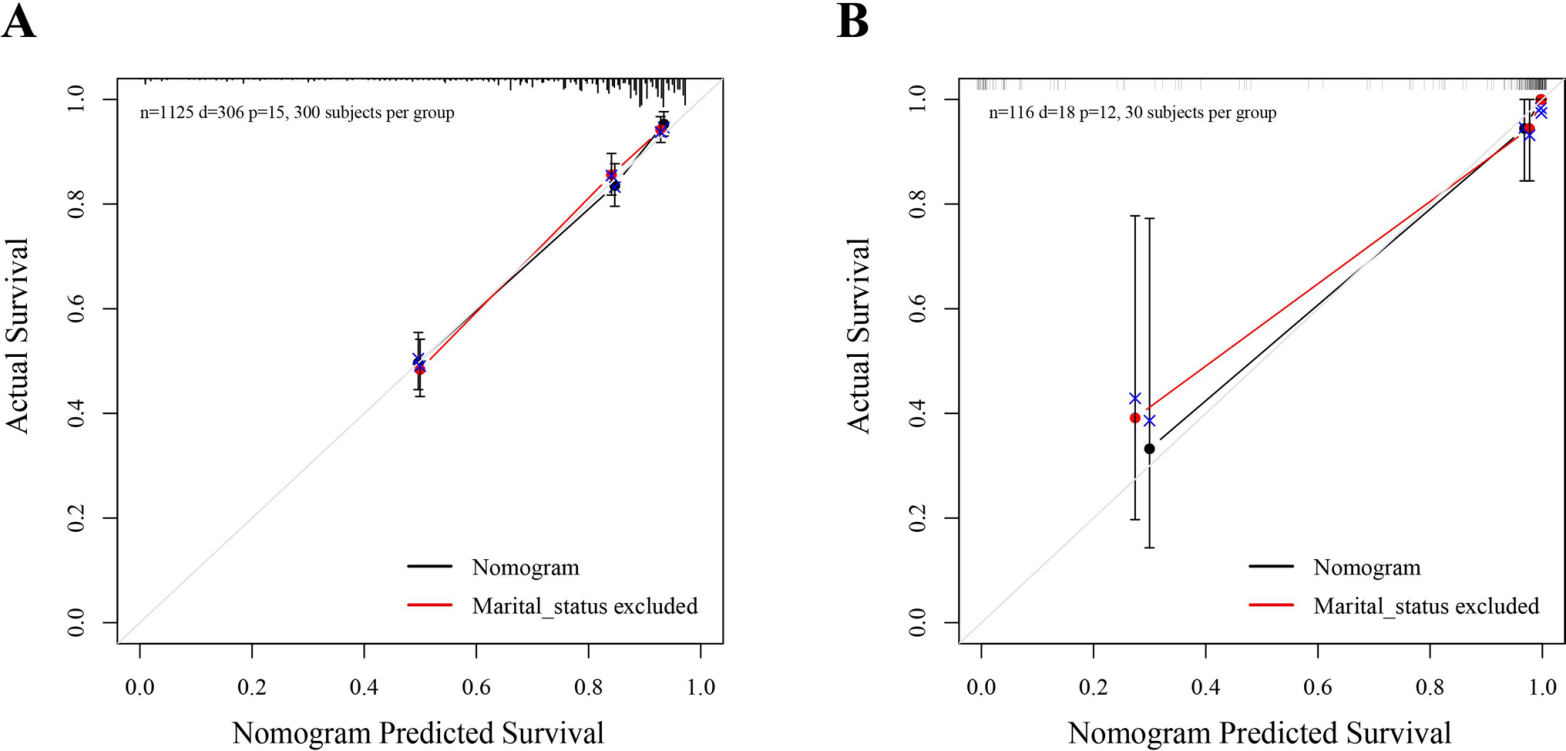
Calibration plots of nomogram. **A** Validated by the training cohort. **B** Validated by the validation cohort

Calibration plots were generated to assess the agreement between the predicted and observed outcomes of the nomogram for both the training cohort and the validation cohort. The plots demonstrated excellent consistency between the nomogram predictions and the actual observations, as shown in Fig. 5. In addition, we observed that excluding marital status weakened the accuracy of the nomogram (Fig. 5B).

**Fig. 6:**
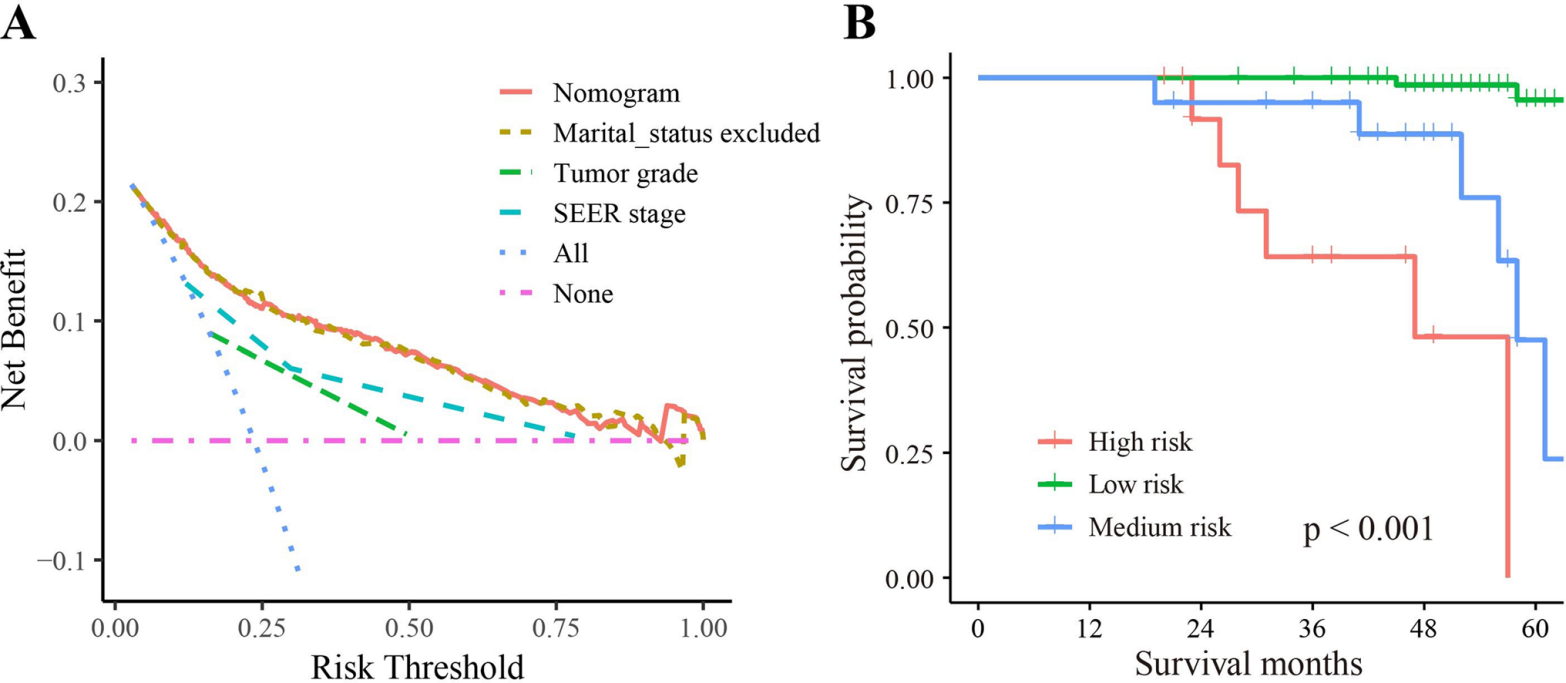
Clinical applicability of nomogram. **A** Decision curve analysis (DCA). **B** Kaplan–Meier survival curve of OS using validation cohort data

DCA was employed to assess the clinical utility of the nomogram. The DCA graph depicted that our nomogram provided the highest net benefit across a wide range (about 0.1 to 1) of reasonable threshold probabilities (Fig. 6A). Importantly, our nomogram exhibited clinical benefit across all ranges of risk thresholds when compared to the model that excluded marital status, highlighting the importance of marital status as a predictive factor.

To further evaluate the discriminatory ability of the nomogram, Kaplan–Meier curves were constructed. The patients in the training cohort were divided into three risk subgroups based on the optimal cutoff values determined through X-tile analysis (low risk: ≤ 135, medium risk: 136–206, and high risk: ≥ 207) according to the nomogram scores. Using the validation cohort data, Kaplan–Meier curves for 5-year OS were plotted, demonstrating the nomogram's ability to accurately differentiate patients at different risk levels, as depicted in Fig. 6B (P < 0.001). The results are summarized in Table 3 and can be further explored in S2 Fig.

**Table 3:** The results of X-tile analysis using training cohort data

Risk stratification	Patients n (%)	Mortality n (%)	Nomogram score	Relative risk
Low risk	743(66.04)	65(8.75)	≤ 135	1 (reference)
Medium risk	265(23.56)	85(32.08)	136–206	3.67
High risk	117(10.4)	91(77.78)	≥ 207	8.89

## Discussion

Marital status has been shown to have a significant impact on survival in various types of cancers, including liver cancer [13], lung cancer [22], gastric cancer [12], kidney cancer [15], melanoma [14], osteosarcoma [23], and CHS [7]. A meta-analysis comprising 67 studies across different cancer types found that unmarried patients, including single, never-married, divorced/separated, and widowed patients, had a higher likelihood of dying from cancer compared to married patients, with increased risk ranging from 17 to 34% [24]. In the case of CHS, Gao et al. reported that divorced and widowed patients had an increased risk of cancer-specific death by 36.7% and 51.6%, respectively, compared to married patients [7].

While metastasis is commonly considered a significant risk factor in CHS patients, the relationship between marital status and metastasis is more intricate. Li et al. found that married CHS patients had an increased risk of pulmonary metastasis (OR = 2.151, 95%CI 0.212–5.207) [8]. Likewise, Hoang et al. reported that being unmarried was associated with a protective factor against distant metastases (OR: 0.268, 95%CI 0.098–0.733) [9]. These findings highlight the complex nature of the relationship between marital status and CHS outcomes.

In this study, we utilized the SEER dataset to examine the differences in OS among CHS patients with different marital statuses. Our findings revealed that the widowed group had the poorest OS. Through multivariate analysis, we identified marital status as an independent prognostic factor for OS in CHS patients (HR = 1.925, 95%CI 1.279 to 2.898, P = 0.002). This suggests that the risk of death for widowed patients may increase by 1.925 times compared to married patients.

Psychologically, a cancer diagnosis can induce more distress compared to other medical conditions [25]. Married patients tend to experience fewer emotional consequences, such as distress and depression, as they are likely to share the emotional burden with their partners [26, 27]. On the contrary, widowed patients often face heightened emotional stress due to the loss of both material and spiritual support provided by their spouse. Additionally, for those who are divorced or separated, the experience of marital dissolution is undoubtedly a stressful event. In contrast, single and married patients have not gone through such stressful circumstances, which may explain the better OS observed in these groups.

Our findings suggest that the influence of marital status on OS is more pronounced in female CHS patients. Interestingly, female patients exhibited a better OS compared to male patients. However, this survival advantage associated with gender was eliminated in divorced/separated patients and widowed patients (Fig. 2). It is worth noting that females tend to gain greater financial benefits from marriage compared to males [28]. As a result, widowed females are more likely to encounter economic difficulties. This financial strain may lead to lower utilization of definitive therapy and an increased likelihood of receiving treatment in healthcare facilities of lower quality. Additionally, females typically have a stronger need for emotional support from their spouse. The presence of love and care can stimulate the release of oxytocin, a hormone that can inhibit the growth of cancer cells through both direct and indirect mechanisms [29]. Consequently, female patients may derive greater benefits from marriage compared to their male counterparts. Furthermore, widowed individuals often exhibit weaker immune responses, characterized by poor lymphocyte response in peripheral blood regions and low activity of natural killer cells [30, 31]. Some evidence suggests that increased social support and a sense of wellbeing can bolster patients' immune systems against cancer [32]. Therefore, it may be beneficial to consider targeted social support services, particularly for widowed females, in the management of CHS patients.

Our study has several strengths. First, our developed nomogram incorporates eight demographic, pathological, and therapeutic variables. Second, to the best of our knowledge, this is the first nomogram that includes marital status as a predictor for 5-year OS in CHS patients. Although the improvement achieved may be relatively modest, this novel inclusion enhances the predictive capability and clinical utility of the model. Third, we used an independent cohort from China, which allows for external validation of our nomogram, thereby indicating its potential applicability in an international context. Fourth, our study encompasses a comprehensive validation process, employing multiple methodologies to ascertain the model's accuracy. Fifth, in our nomogram, tumor stage emerged as the most influential factor in predicting prognosis, which is consistent with previous studies [19]. This alignment in findings bolsters the validity and confidence of our results. Additionally, age, gender, tumor size, histological type, grade, and surgery have also been established as independent prognostic factors for CHS outcomes in previous literature [6, 16, 17, 21]. Given the extensive coverage of these factors in existing research, we will not elaborate on their impact on CHS prognosis in this particular study.

However, it is important to consider several limitations in our study. First, the SEER dataset does not provide information on certain potential prognostic parameters, such as smoking status, alcohol use, and comorbidities. Second, the recording of marital status in the SEER database is only available at the time of diagnosis, and changes in marital status during the course of treatment are not captured. This may affect the interpretation of the results related to marital status. Third, it is challenging to completely eliminate the potential interactions between variables. Marital status, for instance, is often correlated with age, and treatment options are influenced by tumor grade or stage. Fourth, the external validation of our nomogram was conducted using a single-center dataset, which may have limitations such as a relatively small sample size and inherent selection biases. Fifth, due to the limited sample size, we remained unable to ascertain the impact of marital status in the external cohort through survival analyses. Last, although marital status was identified as an independent prognostic factor for OS in CHS patients, its contribution to predicting OS appears modest. The aforementioned limitations may constrain the generalizability of our findings. Therefore, we encourage researchers to conduct more well-designed, multicenter, and prospective clinical studies to validate our model and confirm our conclusions.

## Conclusion

In conclusion, our study revealed significant differences in the OS of CHS patients based on marital status, with widowed patients exhibiting the poorest OS. The influence of marital status on OS is more pronounced in female CHS patients. The OS of both married and single females is significantly higher than that of their male counterparts. However, these findings require further validation in a large independent cohort. Marital status, along with age at diagnosis, gender, tumor size, histological type, tumor grade, SEER stage, and surgical therapy, were identified as independent prognostic factors for OS in CHS patients. Although the contribution of marital status on predicting OS appears modest, our nomogram enables accurate prediction of 5-year OS and facilitates the identification of high-risk patient groups. This nomogram can guide clinicians in optimizing individualized treatments and making informed clinical decisions for CHS patients.

### Supplementary Information

Additional file1 (DOCX 160 KB)

## Data Availability

The SEER datasets used during the current study can be found here: https://seer.cancer.gov/. The ZJCH datasets are available from the corresponding author on reasonable request.
