# Supplementary material for: Development and validation of a nomogram integrating marital status for 5-year overall survival of chondrosarcoma: a population-based study
Source: Discov Oncol. 2024 May 16;15:169. doi: 10.1007/s12672-024-01020-1 (PMC11098994; doi:10.1007/s12672-024-01020-1)
Supplement: Supplementary file 1 — Additional file1 (DOCX 160 KB) [file 12672_2024_1020_MOESM1_ESM.docx]

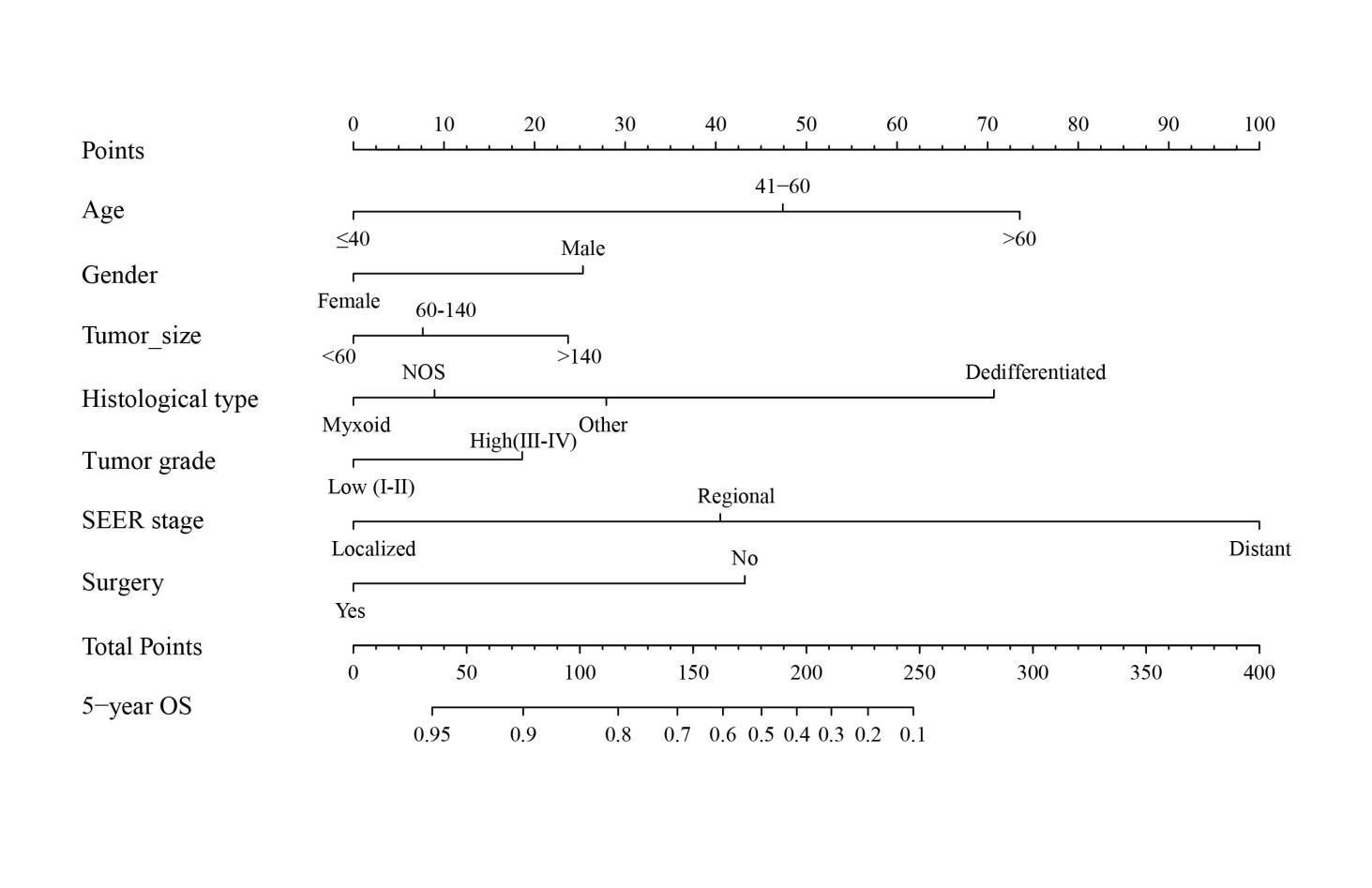


**S1 Fig.** The nomogram did not incorporate marital status as a predictive factor for 5-year OS in CHS patients.


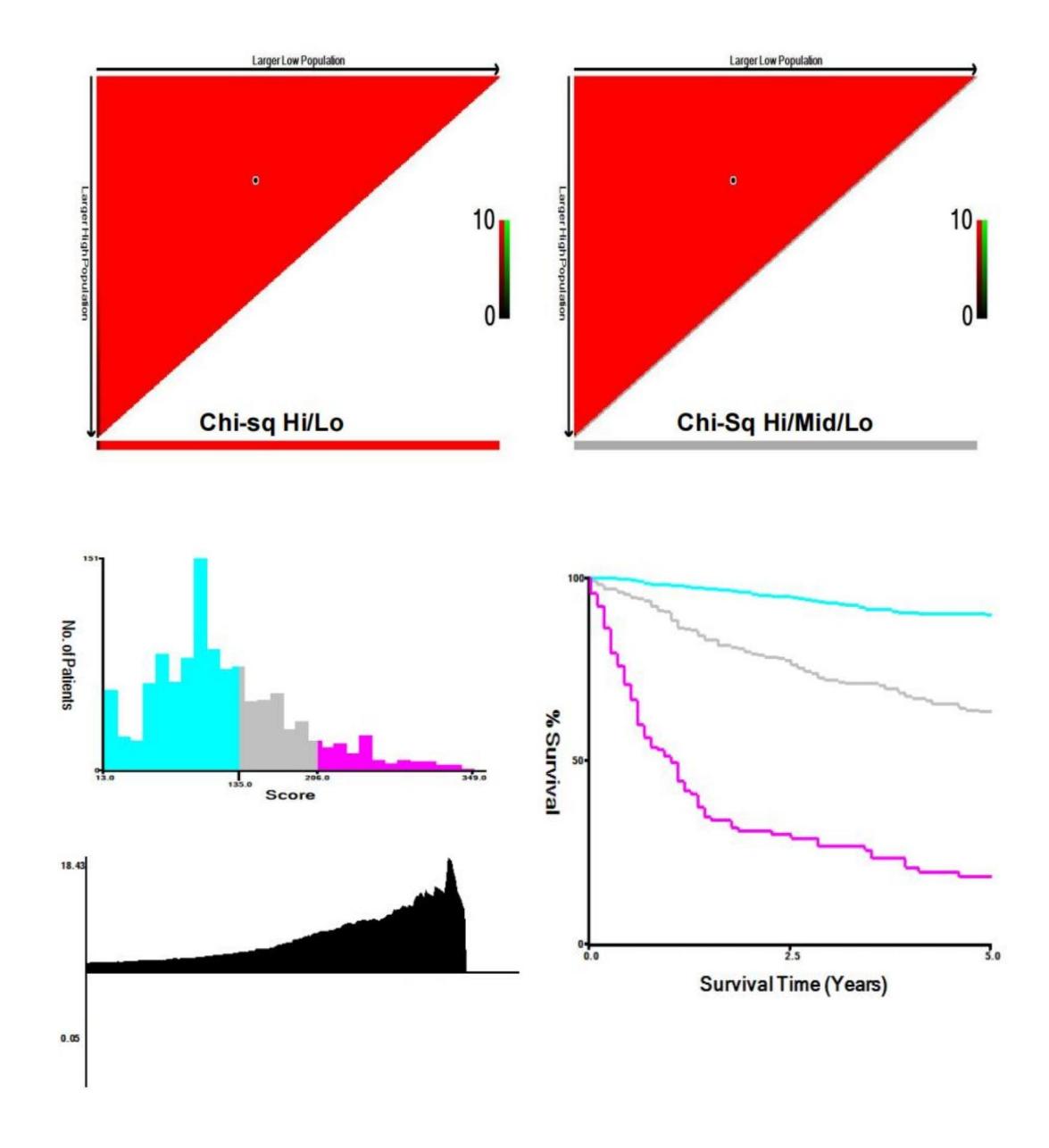


**S2 Fig.** Optimal cutoff values of nomogram score identified by X-tile analysis (training cohort).

| **TABLE S1** \| Baseline characteristics of training cohort and validation cohort | | | |
| --- | --- | --- | --- |
| Variables | Training cohort, n (%) | Validation cohort, n (%) | *P* value |
| Overall | 1125(100) | 116(100) |  |
| Age (years) |  |  | 0.369 |
| ≤40 | 282(25.1) | 35(30.2) |  |
| 41-60 | 464(41.2) | 41(35.3) |  |
| >60 | 379(33.7) | 40(34.5) |  |
| Gender |  |  | 0.516 |
| Female | 511(45.4) | 53(45.7) |  |
| Male | 614(54.6) | 63(54.3) |  |
| Marital status |  |  | 0.001 |
| Married | 724(64.4) | 94(81) |  |
| Div/Sep | 92(8.2) | 10(8.6) |  |
| Single | 253(22.5) | 8(6.9) |  |
| Widowed | 56(5.0) | 4(3.4) |  |
| Race |  |  | <0.001 |
| White | 987(87.7) | 0(0) |  |
| Black | 86(7.6) | 0(0) |  |
| Other | 52(4.6) | 116(100) |  |
| Tumor location |  |  | <0.001 |
| Extremities | 585(52.0) | 26(22.4) |  |
| Axial | 448(39.8) | 66(56.9) |  |
| Other | 92(8.2) | 24(20.7) |  |
| Laterality |  | NC |  |
| Non-paired | 208(18.5) |  |  |
| Left | 450(40.0) |  |  |
| Right | 455(40.4) |  |  |
| Bilateral | 12(1.1) |  |  |
| Tumor size (mm) |  |  | <0.001 |
| <60 | 487(43.3) | 27(23.3) |  |
| 60-140 | 495(44.0) | 76(65.5) |  |
| >140 | 143(12.7) | 13(11.2) |  |
| Histological type |  |  | 0.047 |
| NOS | 910(80.9) | 100(86.2) |  |
| Myxoid | 75(6.7) | 5(4.3) |  |
| Dedifferentiated | 113(10.0) | 5(4.3) |  |
| Other | 27(2.4) | 6(5.2) |  |
| Tumor grade |  |  | 0.001 |
| Low (I–II) | 856(76.1) | 72(62.1) |  |
| High (III– IV) | 269(23.9) | 44(37.9) |  |
| SEER stage |  |  | 0.22 |
| Localized | 631(56.1) | 74(63.8) |  |
| Regional | 400(35.6) | 36(31) |  |
| Distant | 94(8.4) | 6(5.2) |  |
| TNT |  | NC |  |
| 1 | 1034(91.9) |  |  |
| 2 or 3 | 91(8.1) |  |  |
| Surgery |  |  | 0.008 |
| No | 75(6.7) | 16(13.8) |  |
| Yes | 1050(93.3) | 100(86.2) |  |
| Radiotherapy |  |  | <0.001 |
| No | 998(88.7) | 47(40.5) |  |
| Yes | 127(11.3) | 69(59.5) |  |
| Chemotherapy |  |  | <0.001 |
| No | 1036(92.1) | 41(35.3) |  |
| Yes | 89(7.9) | 75(64.7) |  |
| Div/Sep, Divorced/Separated; NOS, not otherwise specified; TNT, Total number of in situ/malignant tumors; NC, Not collected | | | |

| **TABLE S2** \| Nomogram scoring system. | |  |
| --- | --- | --- |
| Variables | Points | |
| Age (years) |  | |
| ≤40 | 0 | |
| 41-60 | 46 | |
| >60 | 68 | |
| Gender |  | |
| Female | 0 | |
| Male | 30 | |
| Marital status |  | |
| Married | 6 | |
| Divorced/Separated | 14 | |
| Single | 0 | |
| Widowed | 42 | |
| Tumor size (mm) |  | |
| <60 | 0 | |
| 60-140 | 9 | |
| >140 | 23 | |
| Histological type |  | |
| Not otherwise specified | 13 | |
| Myxoid | 0 | |
| Dedifferentiated | 72 | |
| Other | 32 | |
| Grade |  | |
| Low (I–II) | 0 | |
| High (III– IV) | 21 | |
| SEER stage |  | |
| Localized | 0 | |
| Regional | 40 | |
| Distant | 100 | |
| Surgery |  | |
| No | 43 | |
| Yes | 0 | |
